# Carbon-coated iron oxide nanoparticles as contrast agents in magnetic resonance imaging

**DOI:** 10.1186/1556-276X-7-44

**Published:** 2012-01-05

**Authors:** Hongsub Bae, Tanveer Ahmad, Ilsu Rhee, Yongmin Chang, Seong-Uk Jin, Sungwook Hong

**Affiliations:** 1 Department of Physics, Kyungpook National University, Daegu, 702-701, Republic of Korea; 2 Department of Molecular Medicine and Diagnostic Radiology, Kyungpook University Hospital, Daegu, 700-422, Republic of Korea; 3 Division of Science Education, Daegu University, Gyeongsan, 712-714, Republic of Korea

**Keywords:** iron oxide nanoparticles, carbon-coated nanoparticles, relaxivity, MRI

## Abstract

Coprecipitated ferrite nanoparticles were coated with carbon using a hydrothermal method. From transmission electron microscope pictures, we could see that the coated iron oxide nanoparticles were spherical in shape with an average diameter of 90 nm. The strong bonding of carbon on the nanoparticle surfaces was checked by noting the C = O and C = C vibrations in Fourier transform infrared spectra. The spin-lattice relaxation process [*T*_1_] and spin-spin relaxation process [*T*_2_] relaxivities of hydrogen protons in the aqueous solution of coated nanoparticles were determined to be 1.139 (mM·s)^-1 ^and 1.115 (mM·s)^-1^, respectively. These results showed that the carbon-coated iron oxide nanoparticles are applicable as both *T*_1 _and *T*_2 _contrast agents in magnetic resonance imaging.

**PACS**: 81.05.y; 76.60.Es; 61.46; 75.50.k; 87.61.

## Introduction

Nanostructured materials have attracted a great deal of attention in the development of biotechnology and medicine [1-3]. Among these nanostructured materials, carbon-coated metal oxide nanoparticles such as MgO, CaO, ZnO, TiO2, Al_2_O_3_, and Fe_2_O_3 _are now extensively studied because of their high application potential [4-9]. Recently, much research interest has been expended on the ferromagnetic iron oxide materials generally used for magnetic data storage as magnetic toners in xerography, and on the ferrofluids, used as contrast agents in magnetic resonance imaging [MRI] [10-13].

The carbon coating provides an effective oxidation barrier and prevents corrosion in magnetic core materials. Hydrophilic carbon coating on iron oxide nanoparticle cores endows better dispersibility and stability than those shown by bare iron oxide nanoparticles. In general, different approaches have been employed for the synthesis of carbon coatings, for example, electric arc discharge, catalytic pyrolysis of organic compounds, and the hydrothermal methods [14].

In this paper, we report the synthesis of carbon coating on iron oxide (Fe_3_O_4_) nanoparticles by a hydrothermal method proposed by Zhang et al. [14] with some modifications. We evaluated these coated particles as potential spin-lattice relaxation process [*T*_1_] and spin-spin relaxation process [*T*_2_] contrast agent in MRI. We studied the *T*_1 _and *T*_2 _relaxations of hydrogen protons in water molecules in an aqueous solution of carbon-coated iron oxide nanoparticles. We found that the *T*_1 _and *T*_2 _relaxivities for the aqueous solution of carbon-coated iron oxide nanoparticles were 1.139 and 1.115 (mM·s)^-1^, respectively. The ratio of these two relaxivities is close to unity. This result demonstrates that carbon-coated iron oxide nanoparticles are suitable as both *T*_1 _and *T*_2 _contrast agents in MRI.

## Methods

### Materials and fabrication

Carbon-coated iron oxide nanoparticles were synthesized by hydrothermal synthetic processes. Bare iron oxide nanoparticles were formed using the coprecipitation method, in which NaOH solution was slowly added to a mixed solution of ferric and ferrous chlorides in a glove box filled with argon gas. During this process, iron oxide nanoparticles were precipitated. These magnetic particles were separated by a magnet and were washed out by methanol, acetone, and DI [deionized] water. The collected nanoparticles were dried in a vacuum oven to obtain a powder sample of nanoparticles for coating. The dried nanoparticles were dispersed in a solution of 0.5 M glucose for 5 h with sonification. During this process, carbon was coated onto the surfaces of the nanoparticles. The solution was dried in a vacuum oven filled with argon gas for 4 h. The dried, coated nanoparticles were redispersed in DI water and filtered through a 100-nm filter paper several times.

### Characterization

The particle size distribution and structure of the carbon-coated nanoparticles were checked using a TEM microscope (TEM, H-7600, Hitachi High-Tech, Minato-ku, Tokyo, Japan). The hydrodynamic diameter and diffusion constant of the coated nanoparticles in water were measured with a dynamic light scattering [DLS] particle size analyzer (ELSZ-2, Otsuka Electronics Co., Ltd., Osaka, Japan). The bonding of carbon onto the iron oxide particles were confirmed by using Fourier transform infrared spectroscopy [FTIR]. For the relaxivity measurements, aqueous solutions of various nanoparticle concentrations were prepared. The concentration of nanoparticles in the aqueous solution was measured with an inductively coupled plasma [ICP] spectrophotometer (IRISAP, Thermo Jarrell Ash, Franklin, MA, USA). The *T*_1 _and *T*_2 _relaxation times of hydrogen protons in the aqueous solution of the coated nanoparticles were measured using an MR scanner (1.5 T Scanner, GE Medical System, Saskatchewan, Canada).

## Results and discussion

**Figure 1:**
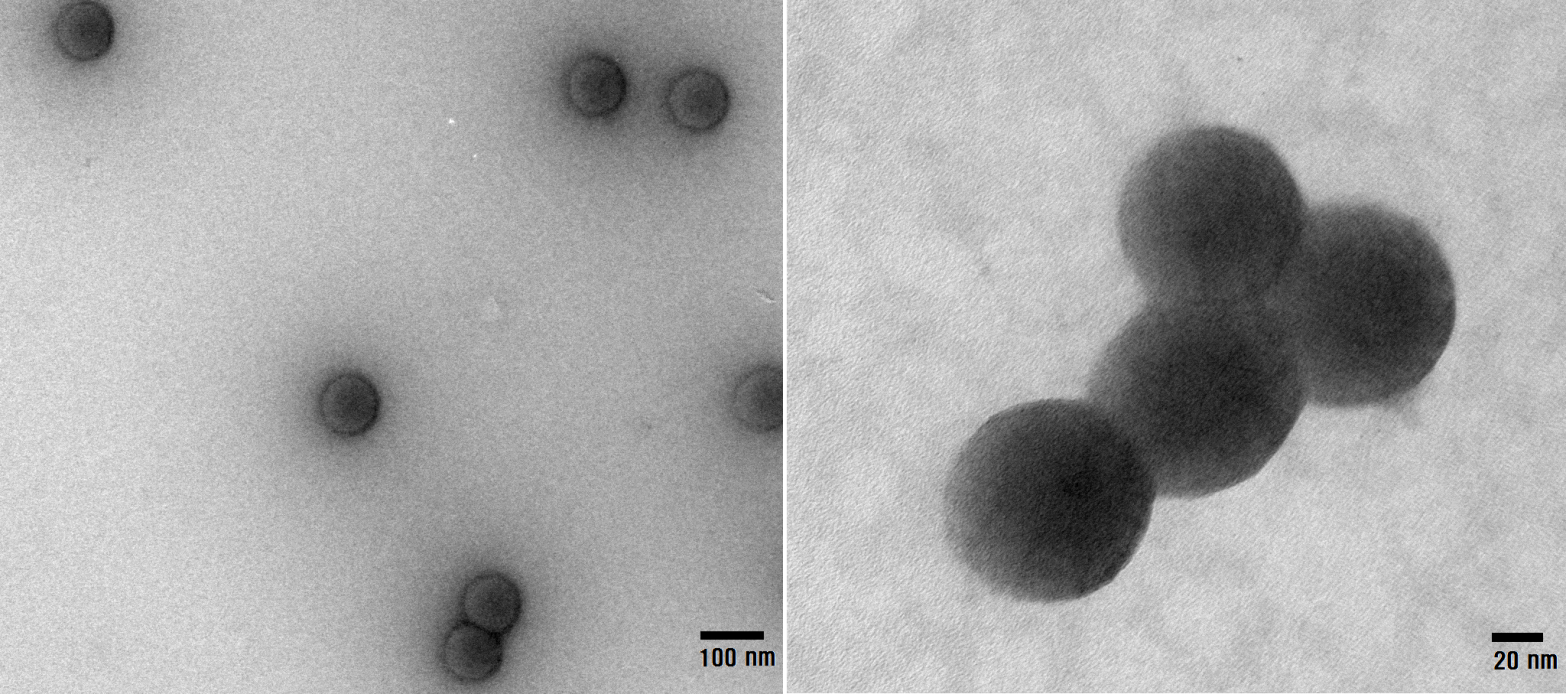
**TEM images of carbon-coated iron oxide nanoparticles**. The coated nanoparticles were spherical in shape with an average diameter of 90 nm.

**Figure 2:**
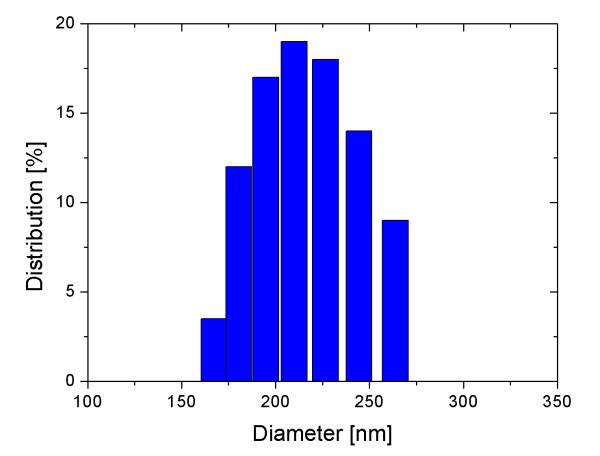
**Particle size distribution**. This figure shows the particle size distribution measured by a DLS particle size analyzer. The average diameter of particles determined from these measurements is about 200 nm. This diameter is larger than the value measured by TEM due to both surface coating of the nanoparticles and the water solvation around the nanoparticles. The diffusion constant of the nanoparticles in water was 9.7265 × 10^-9 ^cm^2^/s.

Figure 1 shows TEM images for the carbon-coated iron oxide nanoparticles. The TEM images showed that the coated nanoparticles were spherical in shape with an average diameter of 90 nm. The average hydrodynamic diameter of the carbon-coated nanoparticles measured by a DLS particle size analyzer was about 200 nm (Figure 2). This diameter was larger than the value measured by TEM due to both surface coating of the nanoparticles and the water solvation around the nanoparticles. The diffusion constant of the nanoparticles in water was 9.7265 × 10^-9 ^cm^2^/s.

**Figure 3:**
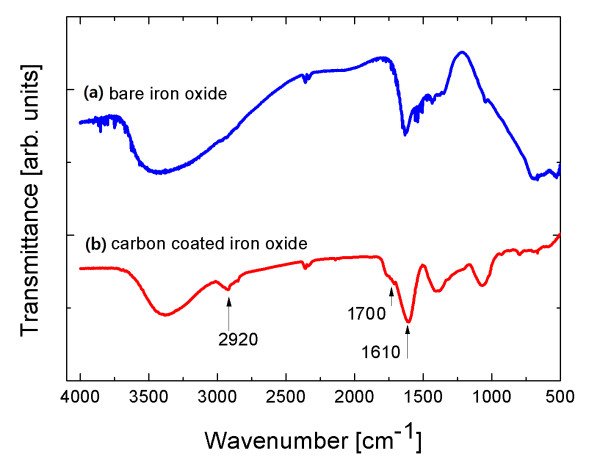
**FTIR spectra for bare iron oxide (a) and carbon-coated iron oxide (b) nanoparticles, respectively**. The bands at 1,700 and 1,610 cm^-1 ^in the spectra of carbon-coated iron oxide are associated with the C = O and C = C vibrations, respectively.

The bonding status of carbon on the surfaces of the nanoparticles was checked using the wavelength-dependent transmittance data obtained using an FTIR spectrometer (Nicolet 380, Thermo Scientific, Waltham, MA, USA). Figure 3a, b shows the FTIR spectra for bare iron oxide and carbon-coated iron oxide nanoparticles, respectively. The bands at 1,700 and 1,610 cm^-1 ^in the spectra of carbon-coated iron oxide are associated with the C = O and C = C vibrations, respectively, which resulted from the carbonization of glucose during the hydrothermal reaction [14,15]. The peaks at 1,000 to approximately 1,400 cm^-1 ^are attributed to the C-OH stretching and O-H bending vibrations. The band at 2,920 cm^-1 ^resulted from the stretching vibrations of O-H.

**Figure 4:**
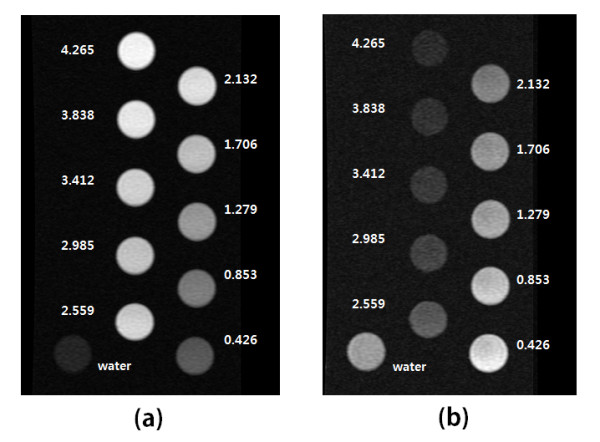
**MR images for T_1 _(a) and T_2 _(b) measurements, respectively**. The circular images in the picture are MR images of aqueous samples of varying concentrations with the units of mM. While a dose-dependent increase in signal intensity is seen in the *T*_1 _measurements, a dose-dependent decrease in signal intensity is observed in the *T*_2 _measurements.

To demonstrate the *T*_1 _and *T*_2 _effects in an aqueous solution, MR images for aqueous solutions of various nanoparticle concentrations were obtained. We prepared ten different samples with varying concentrations (ranging from 0.427 to 4.27 mM of iron) of nanoparticles in DI water and put them into microfuge tubes for imaging. One example of the MR images is shown in Figure 4.

In MRI, the signal recorded during the scan is related to the magnetic relaxation processes of the nuclear spins of the protons in the water molecules in the area of interest. Under a given external magnetic field (*B*), the nuclear spins of the protons align with the field, giving rise to a net magnetization, *M*. If a radio frequency [rf] pulse is perpendicularly applied to *B*, the nuclear spins are excited and start precessing in the plane perpendicular to *B*. Upon removal of the rf pulse, the nuclear spins gradually recover to their equilibrium state parallel to *B*. The recovery of the equilibrium takes place via two different relaxation mechanisms: the spin-lattice relaxation process (*T*_1_), or in other words, the recovery of the magnetization along the *B *direction, and the spin-spin relaxation process (*T*_2_), i.e., the loss of signal in the perpendicular plane. The presence of nanoparticles in the area of interest creates an additional magnetic field (*B*_1_) which induces local field inhomogeneities that significantly increase the speed of the proton transverse relaxation (decrease of *T*_2_), leading to a negative contrast or a darkening of the image.

For *T*_1 _measurements, the inversion recovery pulse sequence was used. In this pulse sequence, the relaxation of nuclear spins in the aqueous solution of the magnetic nanoparticles can be expressed in the following equation:

(1) $$I\sim {\text{M}}_{0}\left(\text{1 }-\text{ 2}e{\;}^{-\frac{t}{T_{1}}}\right)$$

**Figure 5:**
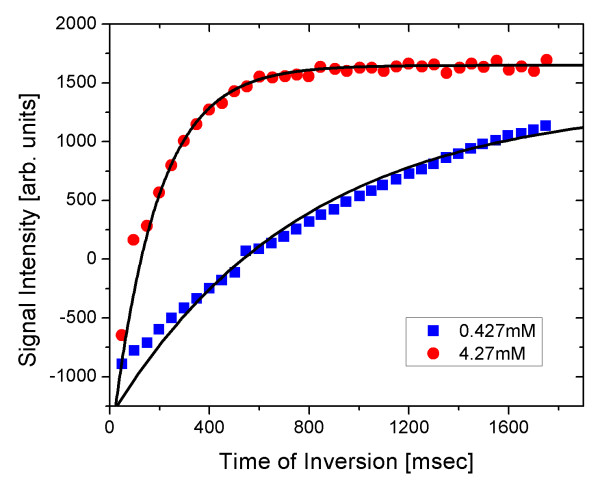
**MR signal intensity for *T*_1 _relaxation**. MR signal intensity as a function of the time of inversion is shown for two representative concentrations of nanoparticles. The *T*_1 _relaxation in the 0.427-mM sample was faster than that of the 4.27-mM sample.

The signal intensities for 35 different times of inversion ranging from 50 to 1,750 ms can be obtained from these MR images. By applying these data to the intensity function of the MR signal, we can obtain the *T*_1 _relaxation time. Figure 5 shows the plot for two different samples of nanoparticles with concentrations of 0.427 and 4.27 mM iron.

The Carr-Purcell-Meiboon-Gill pulse sequence with multiple spin echo was used for the *T*_2 _measurements. MR images for 30 different times of echo ranging from 10 to 1,700 ms were obtained. For the signal intensity function of T_2 _relaxation, the following relationship was used to determine the *T*_2 _relaxation times:

(2) $$I\sim M_{0}e{\;}^{-\frac{t}{T_{2}}}$$

**Figure 6:**
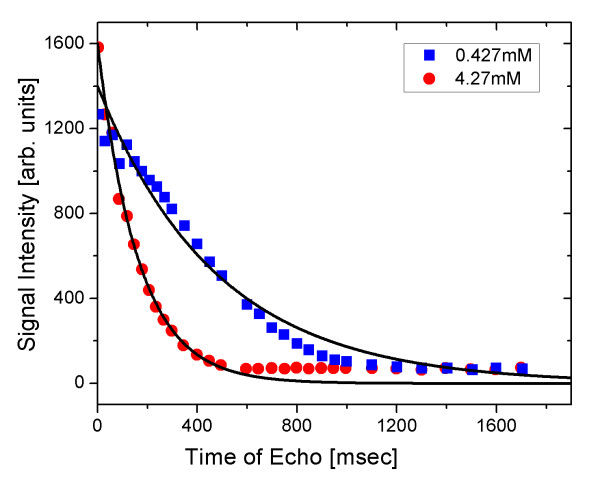
**MR signal intensity for *T*_2 _relaxation**. MR signal intensity as a function of the time of echo is shown for two representative concentrations of nanoparticles. The *T*_2 _relaxation in the 4.27-mM sample was faster than that of the 0.427-mM sample.

Figure 6 shows the plot for two different samples of nanoparticles with concentrations of 0.427 and 4.27 mM iron. The relaxivities $\left(\frac{1}{T_{\text{im}}}\right)$ of nuclear spins in an aqueous solution of magnetic nanoparticles can be expressed as [16]:

(3) $$\frac{1}{T_{\text{im}}}\text{ = }\frac{1}{T_{i}}\text{ + }R_{i}C,$$

where *i *= 1 or 2, and $\frac{1}{T_{i}}$ represents the relaxivity of nuclear spins with no nanoparticle contrast agent. Also, *R*_*i *_is the relaxivity of nuclear spins per mM of nanoparticles, and *C *represents the concentration of nanoparticles in the aqueous solution.

**Figure 7:**
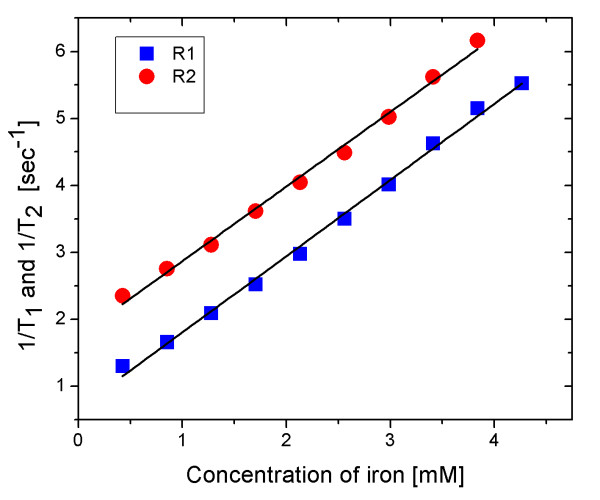
***T*_1 _and *T*_2 _relaxivities**. This figure represents the plots of $\frac{1}{T_{1}}$ and $\frac{1}{T_{2}}$ as a function of the concentration of carbon-coated nanoparticles. The slopes of the straight lines are 1.139 and 1.115 (mM·s)^-1^, respectively.

Figure 7 represents the plots of $\frac{1}{T_{1}}$ and $\frac{1}{T_{2}}$ as a function of the concentration of carbon-coated nanoparticles. The slopes of the straight lines are 1.139 and 1.115 (mM·s)^-1^, respectively. The ratio of these two relaxivities was close to unity. This result demonstrates that carbon-coated iron oxide nanoparticles are suitable as both *T*_1 _and *T*_2 _agents for MRI.

## Conclusions

We synthesized highly water-dispersible carbon-coated iron oxide nanoparticles for use as contrast agents in MRI. The coated nanoparticles were observed to be spherical with a core-shell structure in the TEM images, and they showed a uniform size distribution with an average diameter of 90 nm. The strong bonding of carbon on the nanoparticle surfaces was checked by noting the C = O and C = C vibrations in FTIR spectra. The *T*_1 _and *T*_2 _relaxation times of hydrogen protons were measured using an MRI scanner in the aqueous solutions of various concentrations of nanoparticles ranging from 0.427 to 4.27 mM. The *T*_1 _and *T*_2 _relaxivities were 1.139 and 1.115 (mM·s)^-1^, respectively. The ratio of these two relaxivities was close to unity. This result shows that carbon-coated iron oxide nanoparticles are suitable as both *T*_1 _and *T*_2 _contrast agents in MRI.

## Abbreviations

DLS: dynamic light scattering; FTIR: Fourier transform infrared; ICP: inductively coupled plasma; MRI: magnetic resonance imaging; TEM: transmission electron microscope.

## Competing interests

The authors declare that they have no competing interests.

## Authors' contributions

HB and TA synthesized the carbon-coated nanoparticles. SH carried out FTIR and DLS measurements. YC and SJ performed MRI measurements to determine *T*_1 _and *T*_2 _relaxivities. IR designed the experiments and wrote the manuscript with TA. All authors read and approved the final manuscript.
